# Distinguishing the Rhombohedral Phase from Orthorhombic Phases in Epitaxial Doped HfO_2_ Ferroelectric Films

**DOI:** 10.1021/acsami.4c10423

**Published:** 2024-08-05

**Authors:** Adrian Petraru, Ole Gronenberg, Ulrich Schürmann, Lorenz Kienle, Ravi Droopad, Hermann Kohlstedt

**Affiliations:** † Nanoelectronics, Institute of Electrical Engineering and Information Engineering, Kiel University, Kiel 24143, Germany; ‡ Institute for Materials Science − Synthesis and Real Structure, Faculty of Engineering, Kiel University, Kaiserstraße 2, Kiel D-24143, Germany; § Kiel NanoSurface and Interface Science KiNSIS, Kiel University, Christian-Albrechts-Platz 4, Kiel D-24118, Germany; ∥ Ingram School of Engineering, Texas State University, San Marcos, Texas 78666, United States

**Keywords:** ferroelectric HfO_2_, ultrathin film epitaxy, hafnia, epitaxial Hf_0.5_Zr_0.5_O_2_ thin films, Y-doped ferroelectric HfO_2_.

## Abstract

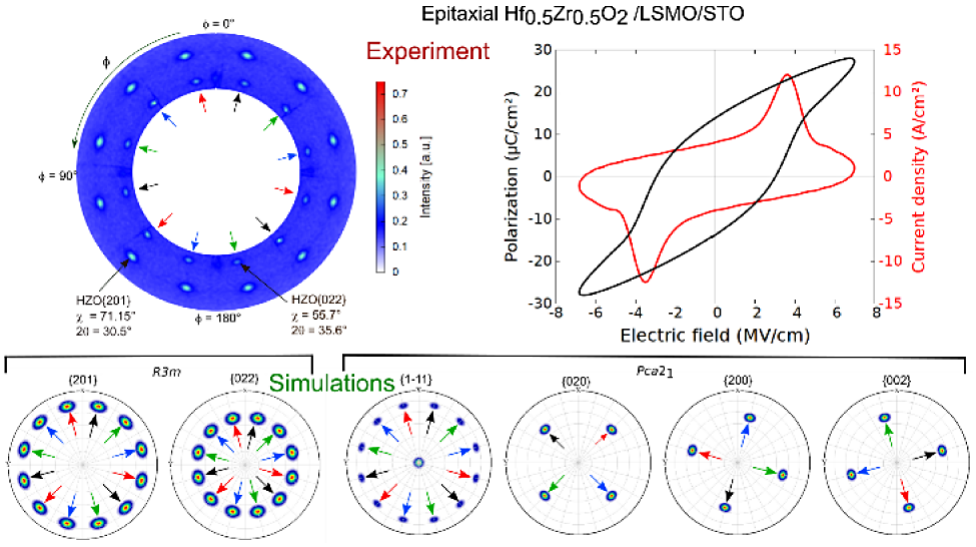

Epitaxial strain plays an important role in the stabilization of ferroelectricity in doped hafnia thin films, which are emerging candidates for Si-compatible nanoscale devices. Here, we report on epitaxial ferroelectric thin films of doped HfO_2_ deposited on La_0.7_Sr_0.3_MnO_3_-buffered SrTiO_3_ substrates, La_0.7_Sr_0.3_MnO_3_ SrTiO_3_-buffered Si (100) wafers, and trigonal Al_2_O_3_ substrates. The investigated films appear to consist of four domains in a rhombohedral phase for films deposited on La_0.7_Sr_0.3_MnO_3_-buffered SrTiO_3_ substrates and two domains for those deposited on sapphire. These findings are supported by extensive transmission electron microscopy characterization of the investigated films. The doped hafnia films show ferroelectric behavior with a remanent polarization up to 25 μC/cm^2^ and they do not require wake-up cycling to reach the polarization, unlike the reported polycrystalline orthorhombic ferroelectric hafnia films.

## 1 Introduction

Ferroelectric materials are of increasing technological importance having continuously extending applications including ferroelectric random-access memories^1,2^ for storage, sensors and actuators,^3,4^ optics,^5,6^ and electronic devices for neuro-inspired electronics such as memristors, necessary for building up electronic synapses for neuromorphic computing.^7−9^ Recently discovered ferroelectricity in doped hafnia ultrathin films opens new perspectives for the realization of special devices like ferroelectric field-effect transistors and ferroelectric tunnel junctions^10,11^ due to their compatibility with the Si technology.

Single-crystal Y-doped HfO_2_ ferroelectric films deposited on yttrium-stabilized zirconia have already been reported, exhibiting the orthorhombic polar phase with the space group *Pca*2_1_ (o-phase) and a polarization of 16 μC/cm^2^, generally considered responsible for ferroelectricity in thin doped hafnia films.^12^ On the other hand, epitaxial Hf_0.5_Zr_0.5_O_2_ films deposited on La_0.7_Sr_0.3_MnO_3_/SrTiO_3_ (substrate) by pulsed laser deposition (PLD) have recently been reported.^13,14^ These films do not crystallize in the commonly reported o-phase, but in a polar rhombohedral phase (r-phase), stabilized via epitaxial strain, and was identified with a large *P*_r_ of 34 μC/cm^2^. Moreover, these rhombohedral films do not require “wake-up” cycling for establishing ferroelectric switching unlike most of the reported polycrystalline films with an orthorhombic polar phase. Recently, polycrystalline Hf(Zr)_1+*x*_O_2_ crystallizing in a rhombohedral ferroelectric phase with a low coercive field have been reported.^15^ Meanwhile, epitaxial doped hafnia films were reported by some authors, being deposited on several single crystal substrates like yttria-stabilized zirconia (111)YSZ, (100)YSZ,^16^ LSMO-buffered LaAlO_3_,^14,17,18^ SrTiO_3_,^13,14,18,19^ NdScO_3_, NdGaO_3_, MgO,^18^ YAlO_3_, (LaAlO_3_)_0.3_–(Sr_2_AlTaO_6_)_0.7_ (LSAT), DyScO_3_,^14,18^ SrTiO_3_-buffered Si,^20^ and GaN-buffered Si.^21^ An interesting study on epitaxial doped hafnia films deposited on various La_0.7_Sr_0.3_MnO_3_ (LSMO)-buffered substrates reveals that TbScO_3_ and GdScO_3_ are very good candidates for epitaxial stress stabilization of the ferroelectric phase.^18^ In most of these studies, the stress-stabilized metastable polar orthorhombic phase (*Pca*2_1_) was reported to be responsible for the ferroelectricity in doped hafnia films,^12,17,19,20,22−26^ whereas the rhombohedral (space groups *R*3*m* or *R*3) phases are less reported. The reason might be the fact that the orthorhombic phase is hard to distinguish from the rhombohedral phase. In this article, we focus on the challenge of distinguishing between these phases.

Apart from the epitaxial hafnia films deposited on sapphire, ITO-buffered YSZ, and GaN, only epitaxial films deposited on LSMO-buffered substrates have been reported to be ferroelectric.

A study on other buffer layers like LaNiO_3_, La_0.5_Ca_0.5_MnO_3_, SrRuO_3_, and Ba_0.95_La_0.05_SnO_3_ showed a low or no stabilized ferroelectric phase, except for those with manganite electrodes.^27^

In addition to the epitaxial strain imposed by the substrate, factors such as deposition temperature, partial gas pressure, and film composition also play an important role in determining the formation of crystal phases. For example, Kaiser et al. reported that either a monoclinic or a rhombohedral phase is stable in HfO_2_ thin films grown by molecular beam epitaxy on c-cut sapphire depending on the oxygen partial pressure. DFT simulations have shown that the rhombohedral phase is stabilized by oxygen vacancies, not due to the epitaxial strain.^28^ Furthermore, it was discussed that a zirconium substitution may have the same effect.

Superlattice and layer structures provide a further improvement of the ferroelectric performance of hafnia-based films. Thus, “wake-up” free ferroelectric capacitors based on HfO_2_/ZrO_2_ superlattices^29^ or capacitors with improved ferroelectric and dielectric performances have been reported.^30,31^

In this work, the focus will be on ferroelectric epitaxial doped hafnia films deposited on three different substrates to compare their structural properties and to determine their crystal structure. Thus, Zr/Y:HfO_2_/La_0.7_Sr_0.3_MnO_3_/SrTiO_3_ (100), Zr/Y:HfO_2_/La_0.7_Sr_0.3_MnO_3_/SrTiO_3_/Si (100), and Zr/Y:HfO_2_/Al_2_O_3_ (0001) systems are investigated here. The structural characterization of each of these three systems (comprising XRD and TEM results) is presented in a separate subsection, and the electrical characterization is shown in the last section of the results.

## 2 Experimental Methods

### 2.1 Materials

Hf_0.5_Zr_0.5_O_2_ and Hf_0.93_Y_0.07_O_2_ thin films of thicknesses varying from 2.5 to 12 nm were deposited by pulsed laser deposition (PLD) on La_0.7_Sr_0.3_MnO_3_-buffered SrTiO_3_ (001) (STO) substrates, La_0.7_Sr_0.3_MnO_3_-buffered Nb (0.5%)-doped SrTiO_3_ (001), SrTiO_3_-buffered Si substrates, and sapphire (0001) substrates. The doped HfO_2_ and the LSMO films were deposited in a single process without breaking the vacuum. The Hf_0.5_Zr_0.5_O_2_-and Hf_0.93_Y_0.07_O_2_-sintered ceramic targets were purchased from EVOCHEM whereas the LSMO target was purchased from PraxAir. A KrF excimer laser of 248 nm in wavelength was used for ablation in a commercial PLD system from Surface GmbH. The La_0.7_Sr_0.3_MnO_3_ films were deposited at a substrate temperature of 780 °C, under 0.15 mbar O_2_, and at a laser fluence and frequency of 1.4 J/cm^2^, and 2 Hz, respectively. The doped HfO_2_ films were deposited at a substrate temperature of 800 °C, under 1.5 × 10^–2^ mbar O_2_, and at a laser fluence of 1.4 J/cm^2^ and a frequency of 5 Hz. After deposition, the films were cooled at 5 C/min to room temperature under 3 mbar of oxygen pressure. Epitaxial 20 nm SrTiO_3_ buffer layers were deposited on Si(100) substrates by molecular beam epitaxy (MBE) at Texas State University. The epitaxial oxide growth on silicon was achieved using a codeposition process in which both the alkaline earth metal and the Ti shutters were opened in a controlled oxygen environment. Since, under these growth conditions, the sticking coefficient of the individual elements is unity, careful calibration of the fluxes was performed for stoichiometric oxide films. The growth rate used was approximately 2A/min and was calibrated using RHEED intensity oscillations.

### 2.2 XRD Characterization

The XRD measurements including wide-range reciprocal space maps (RSMs) and pole figures were acquired with a SmartLab diffractometer (Rigaku) equipped with a 9 kW Cu anode X-ray tube and a 2D HyPix-3000 X-ray detector. A two-bounce monochromator was used for high-resolution XRD scans.

### 2.3 TEM Sample Preparation and Analysis

Cross-sectional TEM samples were prepared from the SrTiO_3_ and Al_2_O_3_ substrates using focused ion beam (FIB) milling in an FEI Helios Nanolab system. Before Ga-ion etching, a protective Pt coating was deposited with a gas injection source inside the FIB system. A plan-view sample on a Si substrate with epitaxial SrTiO_3_ and LSMO layers was prepared with a Precision Ion Polishing System (PIPS, Model 691 from Gatan Inc.).

The FIB samples were analyzed using a JEOL JEM-2100 with electrons accelerated to 200 kV extracted from a LaB6 cathode, while the PIPS sample was analyzed with an FEI Tecnai F30 G^2^ at 300 kV and a field emission gun.

### 2.4 Device Fabrication

Ferroelectric capacitors were fabricated by depositing Cu top electrodes by thermal evaporation through a stencil mask. The device size varies from 25 to 225 μm^2^. Other ferroelectric capacitors on Si substrates were patterned by using UV optical lithography and Ar^+^ ion beam etching.

### 2.5 Electrical Characterization

The ferroelectric hysteresis loops (*P*–*V* loops) of the ferroelectric capacitors were measured by using a Radiant Technologies Premier II ferroelectric tester. The test signal used here consists of a linear ramp waveform with a period in the range of 0.1 to 150 ms. Additional ferroelectric hysteresis loops were measured with an aixACCT TF Analyzer 3000.

## 3 Results and Discussion

### 3.1 Structural Characterization

#### 3.1.1 Hf_0.5_Zr_0.5_O_2_ (HZO) Films Deposited on LSMO-Buffered (001)-Oriented Nb:STO Substrates

##### 3.1.1.1 X-ray Diffraction Analysis

**Figure 1:**
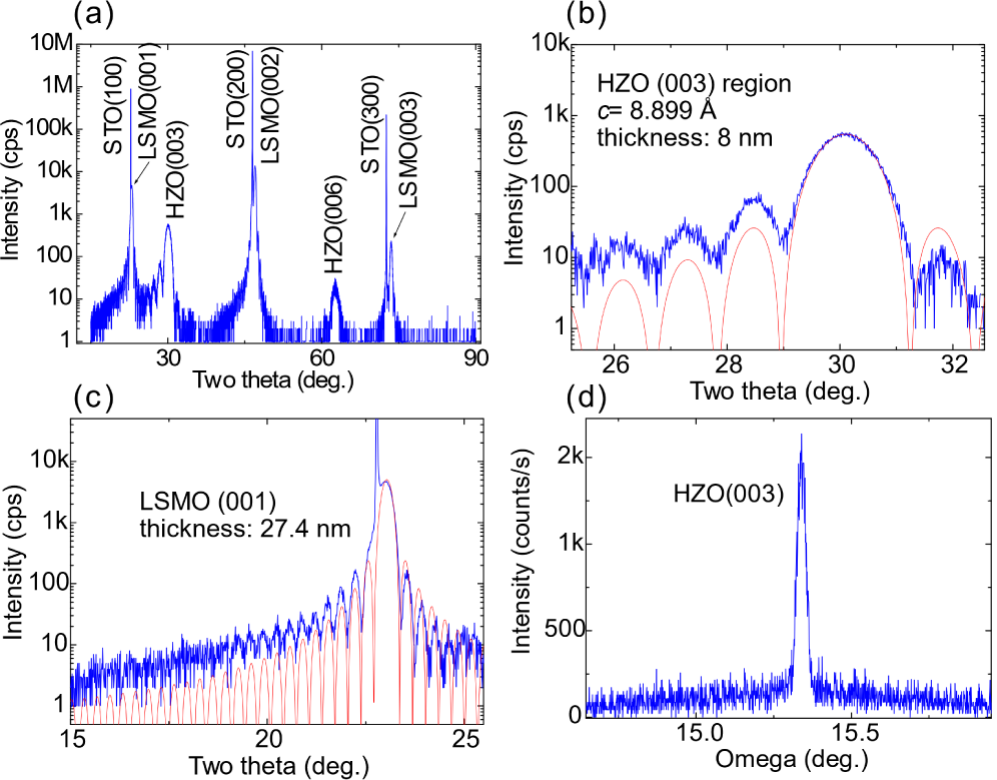
(a) The XRD pattern of the Hf_0.5_Zr_0.5_O_2_/La_0.7_Sr_0.3_MnO_3_/SrTiO_3_ (substrate) heterostructure; (b) the detailed Hf_0.5_Zr_0.5_O_2_ 003 region with thickness oscillations; the blue line represents the experimental data, and the red line is the simulation of the diffractogram showing a film thickness of 8 nm. (c) Simulation of the La_0.7_Sr_0.3_MnO_3_ 001 region (red line), the film thickness is found to be about 71 unit cells, i.e., 27 nm. (d) Rocking curve of the Hf_0.5_Zr_0.5_O_2_ 003 reflection; FWHM = 0.032°.

An X-ray diffraction (XRD) scan of a Hf_0.5_Zr_0.5_O_2_ (HZO) film on LSMO-buffered (001)-oriented Nb:STO substrates is depicted in Figure 1a. The specular reflections 001, 002, and 003 of the Nb:STO substrate are the most intense ones, followed by the specular reflections of the epitaxial LSMO film. The 003 and 006 reflections of the HZO film are also present. Figure 1b shows the magnified 003 HZO region, where the thickness oscillations are clearly visible, demonstrating good crystalline quality and well-defined interfaces. An average HZO film thickness of 8 nm is estimated from the fit of the 003 reflection region, using the formula for the theoretical diffractogram,^32^ adapted for interplanar spacing: , where *d* is the interplanar spacing of the XRD peak involved (the planes are parallel to the sample surface), θ is its corresponding diffraction angle, λ is the X-ray wavelength, and *n* is the film thickness in the number of interplanar spacing. The theoretical diffractogram is plotted with a red line as shown in Figure 1b. A similar analysis for the LSMO film revealed an LSMO film thickness of 27.4 nm, as illustrated in Figure 1c. The rocking curve of the Hf_0.5_Zr_0.5_O_2_ 003 reflection illustrated in Figure 1d shows a FWHM of 0.032°, indicating a good crystalline quality for these films.

**Figure 2:**
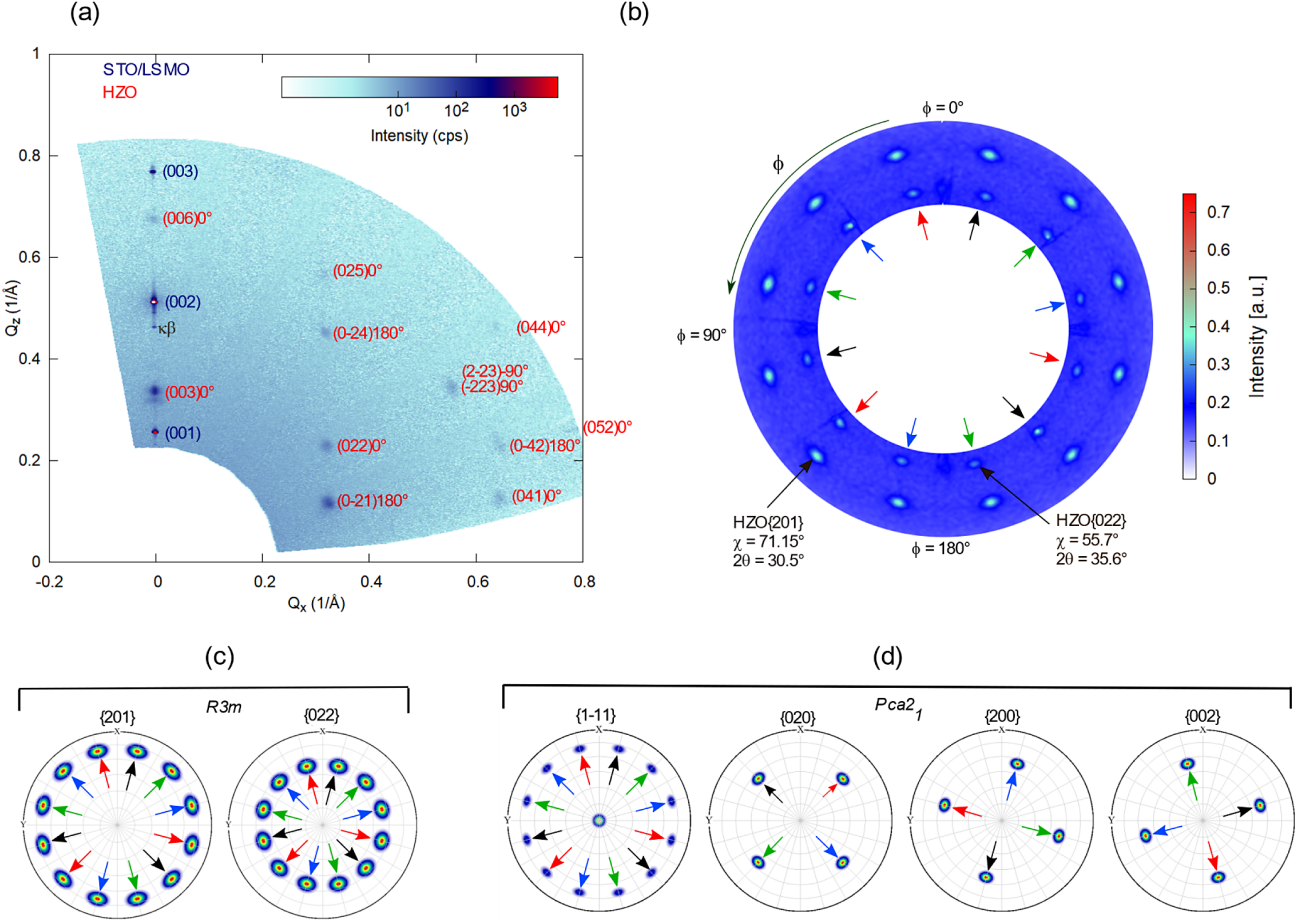
(a) Wide range RSM of the HZO/LSMO/STO (substrate) heterostructure. The spots belonging to LSMO/STO are denoted in dark blue, whereas the spots from the HZO film are denoted in red. The angles marked next to the HZO spots represent the in-plane rotation of the corresponding HZO domains; the *hkl* Miller indices are assigned here to the *R*3*m* phase. (b) Pole figure of the HZO {201} and HZO {022} spots; the black, red, blue, and green arrows indicate the four domains with the families of spots associated with them. Pole figure simulation of the HZO films: (c) phase *R*3*m*, considering (001) out-of-plane orientation and presence of four domains, with an in-plane rotation of 90° with respect to one another; (d) phase *Pca*2_1_ considering (111) out-of-plane orientation and presence of four domains, with an in-plane rotation of 90° with respect to one another, and the presence of {200}, {020}, and {002} spots.

Next, wide-range reciprocal space maps (RSMs) and pole figures were measured for the same stack to determine the crystallographic phase and the orientation of the HZO films. Thus, in the reciprocal space map of the Hf_0.5_Zr_0.5_O_2_/La_0.7_Sr_0.3_MnO_3_/SrTiO_3_ (substrate) heterostructures shown in Figure 2a, the substrate reflections 001, 002, and 003 are identified and labeled in dark blue color on the RSM. The reciprocal space spots corresponding to the epitaxial LSMO layer could not be distinguished from those of the STO substrate, due to lack of resolution in the wide range RSM measurements. Here, we like to specify that the sample was on purpose in-plane rotated by an angle φ = 15° (with respect to the in-plane *a*-direction of the STO substrate) to avoid the contribution of nonspecular spots of the STO/LSMO). For more clarity, a wide-range RSM of the same sample measured at φ = 45° is shown in Figure S1, where the 111, 112, 113, and 221 spots of the STO/LSMO are also present. The HZO films grow epitaxially out-of-plane on the LSMO/STO template, and the films appear to consist of four kinds of domains, belonging to the same crystallographic *R*3*m* phase, first reported by Wei et al.,^13^ having four kinds of in-plane orientations, rotated by 90° with respect to one another. A similar domain structure was reported by Nukala et al. for such heterostructure.^14^ The reason for this domain configuration could be explained by the 4-fold symmetry imposed by the substrate, as also described by Estandía et al.^18^ and observed in heteroepitaxy of oxides^33^ or semiconductors.^34^ The results are illustrated in Figure 2a. The angles marked next to the HZO spots in the wide-range RSM represent the in-plane rotation of the corresponding HZO domains. From the 022 HZO spot, we extracted the lattice parameter values of *a* = 7.17 and *c* = 8.82 Å. More precise values will be extracted from the high-resolution in-plane and out-of-plane scans.

**Figure 3:**
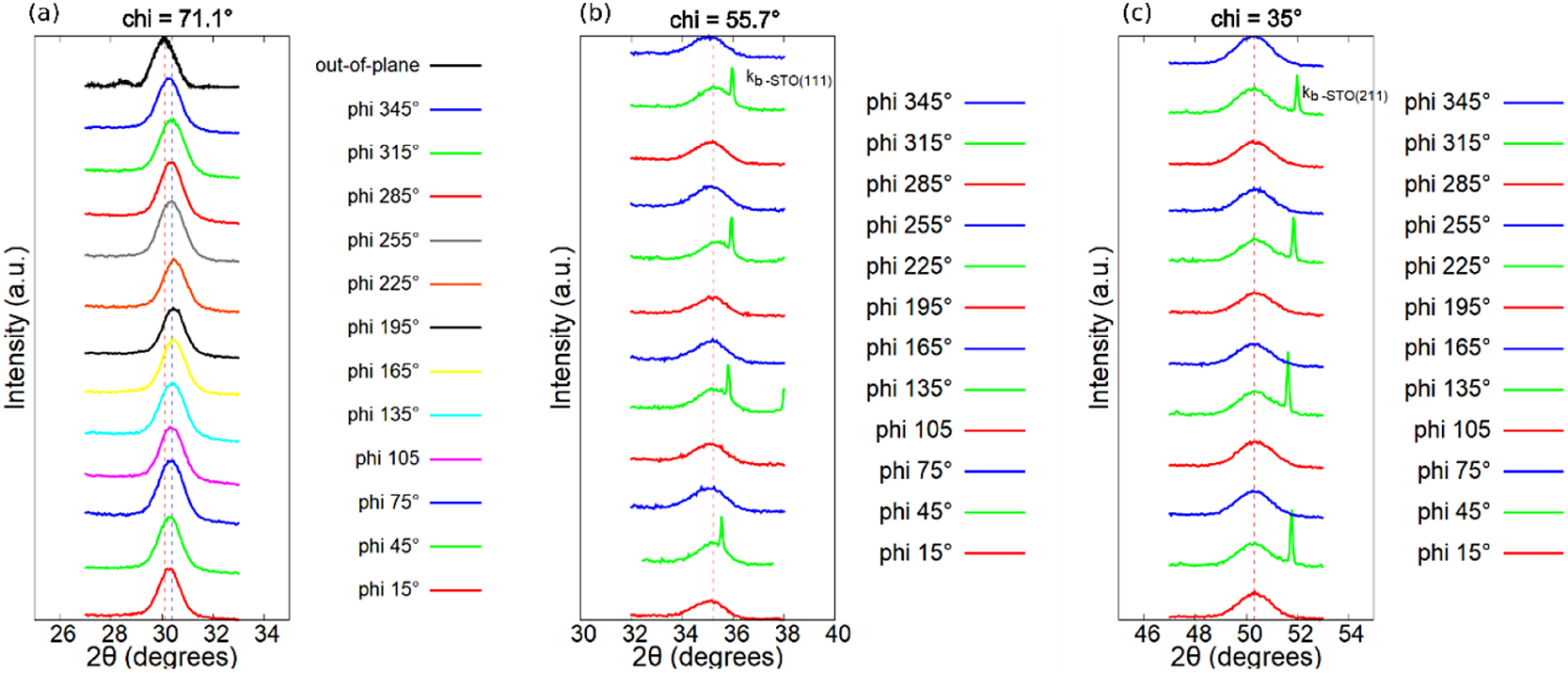
HZO/LSMO/STO (substrate) heterostructure. (a) 2θ scans of the 12 poles at chi = 71.1° and of the specular spot (chi = 0° in black). 2θ scans of the 12 poles at (b) chi = 55.7° and (c) at chi = 35°.

A pole figure of the HZO/LSMO/STO (substrate) heterostructure is shown in Figure 2b. The radial direction represents χ, which ranges between 51° and 85°, while the azimuthal direction represents φ, which ranges between 0° and 360°. Please note that the pole figure presented here is not measured for a single reflection but for a two-theta range between 23° and 37°. Thus, the HZO {201} and HZO {022} families of reflections are seen here. The 12 poles of the HZO {201}, measured at a 2θ angle of 30.5° and the 12 poles of the HZO {022} measured at a 2θ angle of 35.6° are clearly visible in the pole figure. From the crystal symmetry of the *R*3*m* phase, one expects three poles for each, spaced by 120°, but the presence of four kinds of crystallographic domains rotated 90° with respect to one another would give us the observed 12 poles. To reproduce the measured data, we considered four domains of the *R*3*m* phase having the (001) orientation and an in-plane rotation of 90° with respect to one another and simulated the pole figure using the MTEX^35^ simulation tool. The simulated pole figure is presented in Figure 2c. As can be seen, both {201} (at chi = 71.15°) and {022} (at chi = 55.7°) spot families could be reproduced in the simulation of the *R*3*m* phase. If one considers the orthorhombic *Pca*2_1_ phase with a single (111)-oriented domain, three poles rotated by 120° are expected for the HZO{1}, from the crystal symmetry, as simulated with MTEX and also experimentally reported,^18^ and one pole for each of the {020}, {200}, and {002} reflections. The HZO{1} of the *Pca*2_1_ phase would be the corresponding HZO{201} spots of the *R*3*m* phase, and the HZO{020} of the *Pca*2_1_ phase would be the corresponding HZO{022} spots of the *R*3*m* phase. Furthermore, if one considers (111) grown films consisting of four domains having four in-plane orientations rotated 90° with respect to one another and simulates the pole figure, one can reproduce the 12 spots corresponding to HZO {1}, as shown in the simulated pole figure from Figure 2d. To enhance clarity, the poles from each domain in the simulation from Figures 2c,d are indicated with an arrow of a specific color. Thus, we have black, red, blue, and green arrows to indicate the four domains. Additionally, 12 poles are expected at a two-theta angle of about 35.6°, each of the {020}, {200}, and {002} reflections contributing with four spots in the simulation, separated by a 90° angle in phi. The corresponding two-theta angles of the nonsymmetry equivalent 020, 002, and 200 reflections of the *Pca*2_1_ phase are close to each other. Thus, it is difficult to distinguish between the *R*3*m* and *Pca*2_1_ phases based on the (low resolution) wide-range RSM and pole figures. One way to distinguish the *R*3*m* phase from the *Pca*2_1_ phase is to compare the interplanar spacing of the 12 spots measured at a chi angle of 71.15° with the interplanar distance of the specular 111 spot of the *Pca*2_1_ phase. In the case of the *Pca*2_1_ phase, all spots should have the same *d*-spacing (due to the symmetry), whereas for the *R*3*m* phase, the specular spot (the 003 reflection) should have a larger *d*-spacing value.^13,14^ Symmetric two-theta scans of the 12 poles measured at chi = 71.15° are plotted together with the out-of-plane reflection (chi = 0°) as shown in Figure 3a. The 12 spots share nearly the same 2θ values, whereas the out-of-plane reflection has a smaller 2θ (larger *d*-spacing), which is an indication for the *R*3*m* phase.

Another possibility to distinguish between the two polymorphs is to measure symmetric 2θ scans at around 35° for the 12 spots at a chi angle of 55.7°. In the case of the *R*3*m* phase, the {022} spots should have the same 2θ values, whereas for the *Pca*2_1_ phase, the {020}, {200}, and {002}, each contributing with four spots (from the four domains) separated by 90° in φ with respect to one another (see Figure 2d), should have slightly different 2θ values. Similarly, one can analyze the 12 spots measured at chi = 35° at a 2θ of about 50°. Thus, for the *R*3*m* phase, the {204} spots should show the same 2θ values whereas for the *Pca*2_1_ phase, the {022}, {202}, and {220} each contributing with four spots separated by 90° in φ with respect to one another should have slightly different 2θ values. The symmetric two-theta scans of the 12 poles measured at chi = 55.7° and a 2θ value around 35° are presented in Figure 3b, and the 12 poles measured at chi = 35° and a 2θ value of around 50° are shown in Figure 3c. To improve the ease of understanding for the reader, the 2θ scans of each {020}, {200}, and {002} as in Figure 3b and {022}, {202}, and {220} reflections as in Figure 3c corresponding to the *Pca*2_1_ phase are depicted using distinct colors: red, green, and blue. Thus, in the case of the *Pca*2_1_ phase, the reflections of the same color (separated in phi by 90°) should share the same 2-theta value and should be slightly different for each color. When these 2θ values are all equal, assuming the ferroelectric *Pca*2_1_ phase, would imply *a* = *b* = *c*, which contradicts the reported metrics of this crystallographic phase, making it unlikely that the investigated films are in the *Pca*2_1_ phase. These results are instead in good agreement with the metrics of the *R*3*m* phase. However, the existence of a separate ferroelectric rhombohedral phase which is not just a structural distortion caused by epitaxial stress distortion of the *Pca*2_1_ orthorhombic phase is still under debate, as suggested by Fina and Sánchez.^36^

To gain a clearer understanding of how the scans from Figure 3c relate to particular reflections and domains, simulated pole figures for both *R*3*m* and *Pca*2_1_ phases of the 12 spots at chi = 35° and a 2θ of about 50° are shown in Figure S5.

##### 3.1.1.2 Transmission Electron Microscopy Characterization

**Figure 4:**
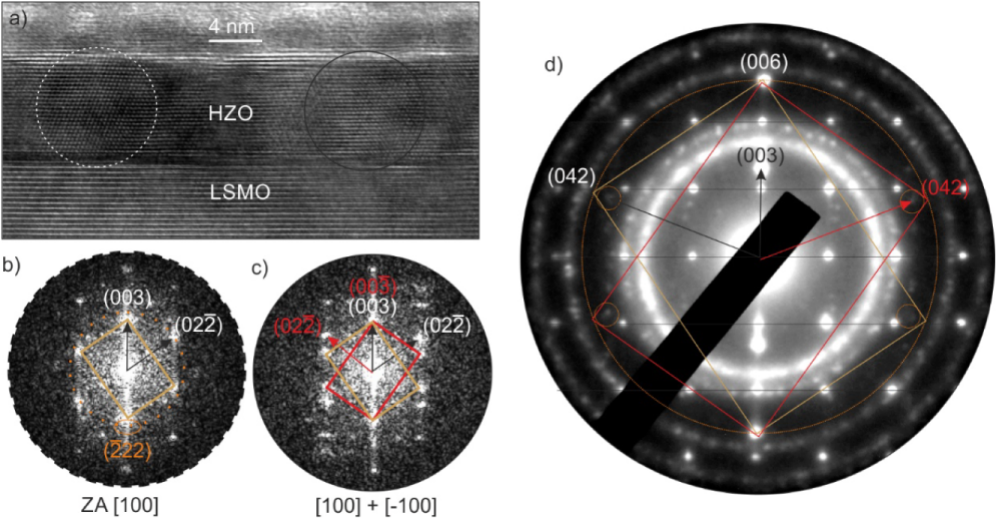
(a) HRTEM micrograph showing an LSMO back electrode in ZA [2] growing along (012) planes. An FFT analysis shown below in (b) and (c) was performed on the two HZO regions marked with circles. The left grain is in [100] ZA, and the right FFT shows a superposition of a [100] and a [00] ZA which could be explained by a twin with a 2-fold rotation at the [003] direction. These domains are indicated by yellow and red rectangles. The orange ellipse in the left FFT in (b) marks the additional [22] minority growing directions to the main [003] growing direction. The SAED pattern in (d) is similar to that of FFT in (c).

The same HZO film on the LSMO-buffered (001)-oriented Nb:STO substrate analyzed by XRD was used for TEM characterization. The HRTEM micrograph in Figure 4a shows the LSMO back electrode growing along (012) planes while the HZO thin film grows mainly in the [003] direction of the *R*3*m* phase. However, a minority growing direction along [22] was also observed (marked with an orange ellipse in the FFT in Figure 4b, as also reported by Wei et al).^13^ This minority growth direction is not observed in XRD measurements. Different domains of the HZO were present with (02) planes oriented to the right or the left, these planes are indicated by a black and a red arrow, respectively. Frequently both orientations superimpose, as can be seen in the FFT in Figure 4c. This pattern can be explained by a 2-fold twin rotation around the [003] growing direction. Accordingly, we can see here at least two of the four domains observed in XRD. The missing two domains cannot be distinguished in this orientation because they have the same diffraction pattern. For instance, the [110] zone axis (ZA) is equivalent to the [100] ZA in a rhombohedral symmetry. A clear view of the boundary of the domains, however, was not found in many micrographs examined.

The selected area electron diffraction (SAED) pattern in Figure 4d was aligned parallel to the [100] ZA of the STO. In this orientation, the LSMO is in the [2] ZA and the epitaxial growth of all films is clearly visible. The diffraction patterns of STO and LSMO are indicated by black horizontal lines. For HZO, the (003) reflections in the growing direction are most prominent, while in-plane no reflections can be evidenced, and also the diagonal planes are very faint (marked with orange circles).

Furthermore, from this SAED pattern, a difference in the d-values of (006) and (042) can be observed, as illustrated by the dashed ring around the (006) reflections. These reflections would correspond to the {222} planes of the ferroelectric orthorhombic phase, which cannot have a *d*-value difference (in a relaxed structure) due to the orthorhombic symmetry. An effect of an elliptical distortion of the diffraction pattern by unprecise adjustments of the projector lens system can be ruled out, because the reflections of the polycrystalline Pt from the FIB preparation are not elliptically distorted.

Superimposed are the [100] ZA of STO and the [2] ZA of LSMO which are illustrated by black vertical lines. The reflections of HZO in the diagonal are very faint. Therefore, the rectangles from the FFT are used here double sized to illustrate the diffraction pattern of HZO. The rings in the SAED pattern in (d) originate from the protective Pt coating used in the FIB.

#### 3.1.2 Hf_0.93_Y_0.07_O_2_ (HYO) Films Deposited on SrTiO_3_-Buffered Si (100)-Oriented Substrates

##### 3.1.2.1 X-ray Diffraction Analysis

**Figure 5:**
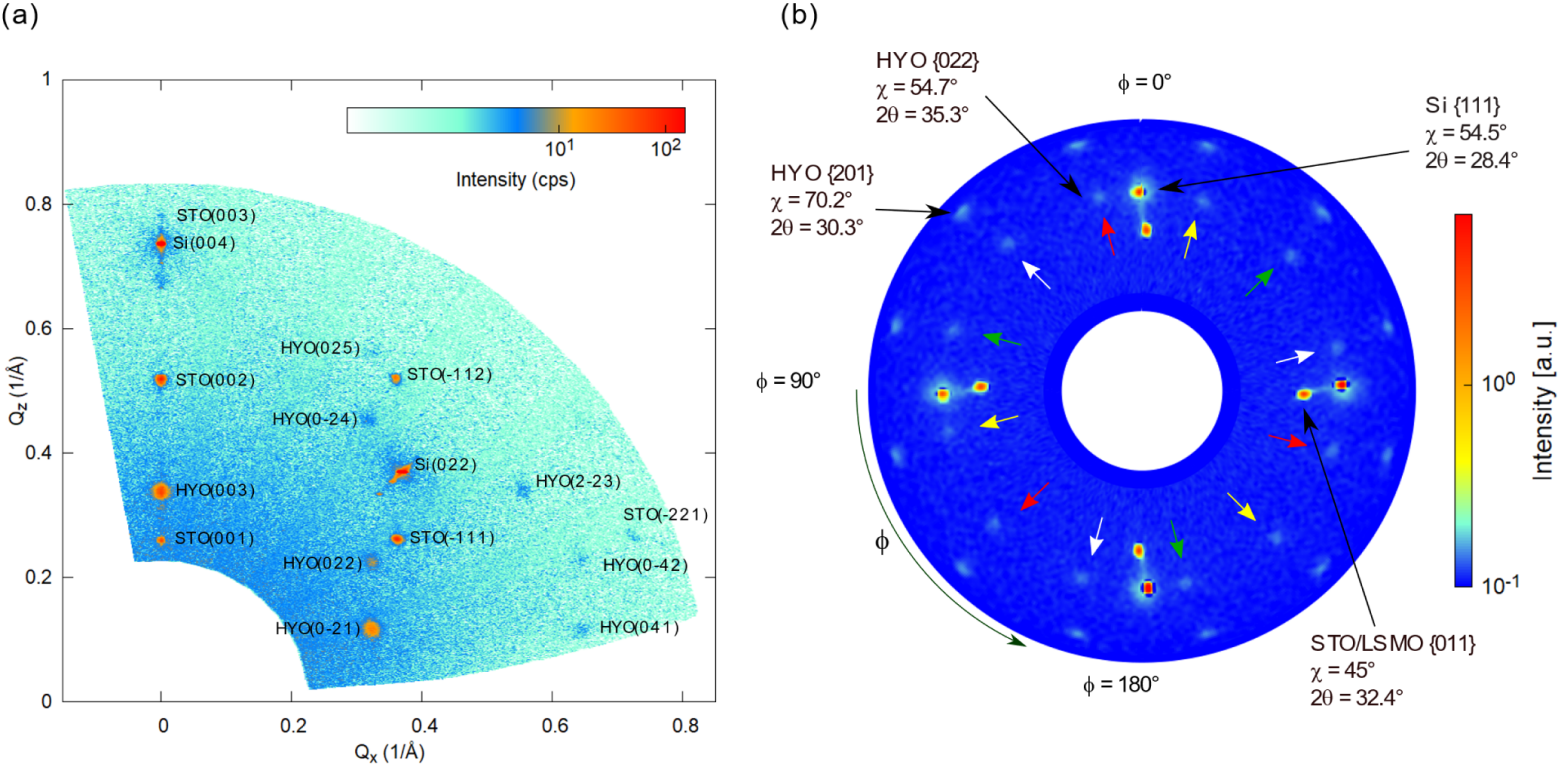
(a) Wide-range RSM of a 5.6 nm thick HYO film deposited on La_0.7_Sr_0.3_MnO_3_ /SrTiO_3_/Si (substrate); here, the observed spots from the HYO film are assigned to (001)-oriented films of the ferroelectric *R*3*m* phase, consisting of four types of domains with 0°, 90°, 180°, and 270° in-plane orientation. The measurement was acquired at φ = 0°. (b) Pole figure of the HYO {022}, HYO {201}, STO/LSMO {011}, and Si {111} reflections; the yellow, red, white, and green arrows indicate the four domains with the families of spots associated with them. The measured 2θ range is 24.9–37.4°. The radial direction represents the χ axis ranging from 0° to 90°, whereas the azimuthal direction represents the φ axis with a range from 0° to 360°.

Yttrium-doped Hafnia films Hf_0.93_Y_0.07_O_2_ (HYO) were deposited on Si (100)-oriented substrates on which a 10 nm SrTiO_3_ (STO) was deposited by molecular beam epitaxy (MBE) at Texas State University.^37,38^ The Hf_0.93_Y_0.07_O_2_/La_0.7_Sr_0.3_MnO_3_ epitaxial stack was deposited by PLD, using the same deposition parameters as those deposited on Nb:SrTiO_3_ substrates. The wide-range RSM of the doped hafnia films deposited on La_0.7_Sr_0.3_MnO_3_ /SrTiO_3_/Si (substrate) shows features similar to those deposited on La_0.7_Sr_0.3_MnO_3_ /SrTiO_3_ (substrate), an out-of-plane orientation, and the presence of four kinds of domains with different in-plane orientations, as described before. The wide-range RSM was acquired at φ = 0°, and besides the specular Si(004), the Si(022) spot was visible in this diffraction geometry. The nonspecular STO/LSMO diffraction spots 11, 12, and 21 are also present. The results are shown in Figure 5a. A pole figure of HYO{022}, HYO{201}, STO/LSMO{011}, and Si{111} measured for a 2θ ranging from 34.8° to 47.8° is shown in Figure 5b. The 12 spots of the HYO{022} and HYO{201} arising from the four domains are present on the pole figure, similar to the results from doped hafnia films deposited on LSMO-buffered STO substrates.

##### 3.1.2.2 Transmission Electron Microscopy Characterization

**Figure 6:**
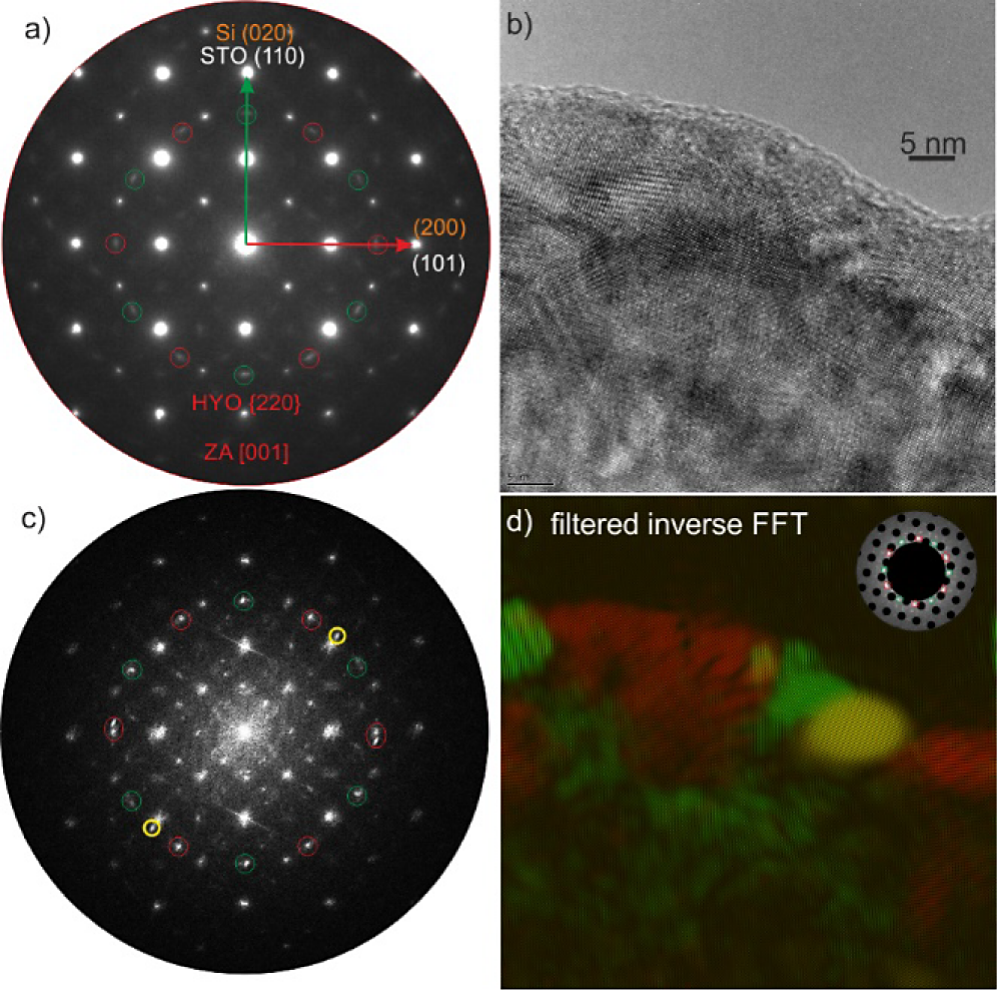
(a) Plan-view SAED pattern of Si/STO/LSMO/HYO. The substrate Si is oriented in the [100] zone axis as well as the STO which is rotated by 45° with respect to Si. HYO shows mainly two types of reflections. The red circles depict the HYO [001] zone axis with {220} reflections aligned with the STO (101) planes (red arrow) while the reflections marked by the green circles are aligned with the STO (110) planes (green arrow). (b) shows a high-resolution TEM micrograph in the same viewing direction which is confirmed by the FFT in (c) showing the same diffraction pattern as in a). In (c), the green, red, and yellow circles were used for filtering the FFT and building a colored inverse FFT shown in (d). The majority of the domains have an [003] out-of-plane orientation with {220} reflections marked by red and green circles. The minority (yellow) is oriented with STO (200).

The TEM preparation of plan-view samples grown on the STO substrates was not successful because in 3 trials the samples broke apart during mechanical polishing before finishing the preparation. As a result, a Zr/Y:HfO_2_ sample on SrTiO_3_-buffered Si was prepared. This sample should be representative, as indicated by the XRD analysis. A SAED pattern of the plan-view sample with Si oriented in the [100] ZA is shown in Figure 6a. Here, the HYO forms a similar diffraction pattern as reported by Wei et al.^13^ with 12 {120} reflections which can be explained by HYO having at least two domains rotated in plane by 90°. The green and red circles in Figure 6a indicate two domains. The domains marked with green circles are oriented with the STO (110) planes while the domains marked with red circles are oriented with the STO (101) planes. Adding the 2-fold twin rotation observed in the cross-sectional TEM analysis results in the four domains observed in XRD pole figures. These four types of domains may form during the crystal nucleation during PLD, where each nucleus can be oriented with STO (110), (−110), (1–10), or (−1–10). The same color code is used in the FFT shown in Figure 6c of the HRTEM micrograph in shown Figure 6b. These colored circles were used for the filtered inverse FFT in Figure 6d which illustrates the granular microstructure of the HYO thin film. However, all individual grains are epitaxial.

A TEM image in a different orientation, tilted away from the [100] zone axis of Si, is shown in Figure S4. Here, strong Moiré fringes appear which were again used for a filtered inverse FFT. From these false color images, the average width of 20 domains can be roughly estimated to be 10 nm ±3 nm. The high crystallinity of the HZO, as indicated by the rocking curve, argues for coherent interfaces between these 10 nm-sized domains as would be the case for twins.

In addition to the observed majority reflections in the SAED pattern shown in Figure 6a from HYO with the [003] out-of-plane orientation, a minority is observed in the FFTs (see yellow circles in Figure 6c and blue circles in Figure S4b) that is oriented with the STO (200) planes.

#### 3.1.3 Hf_0.5_Zr_0.5_O_2_ Films Deposited on Al_2_O_3_ (0001) Substrates

##### 3.1.3.1 X-ray Diffraction Analysis

**Figure 7:**
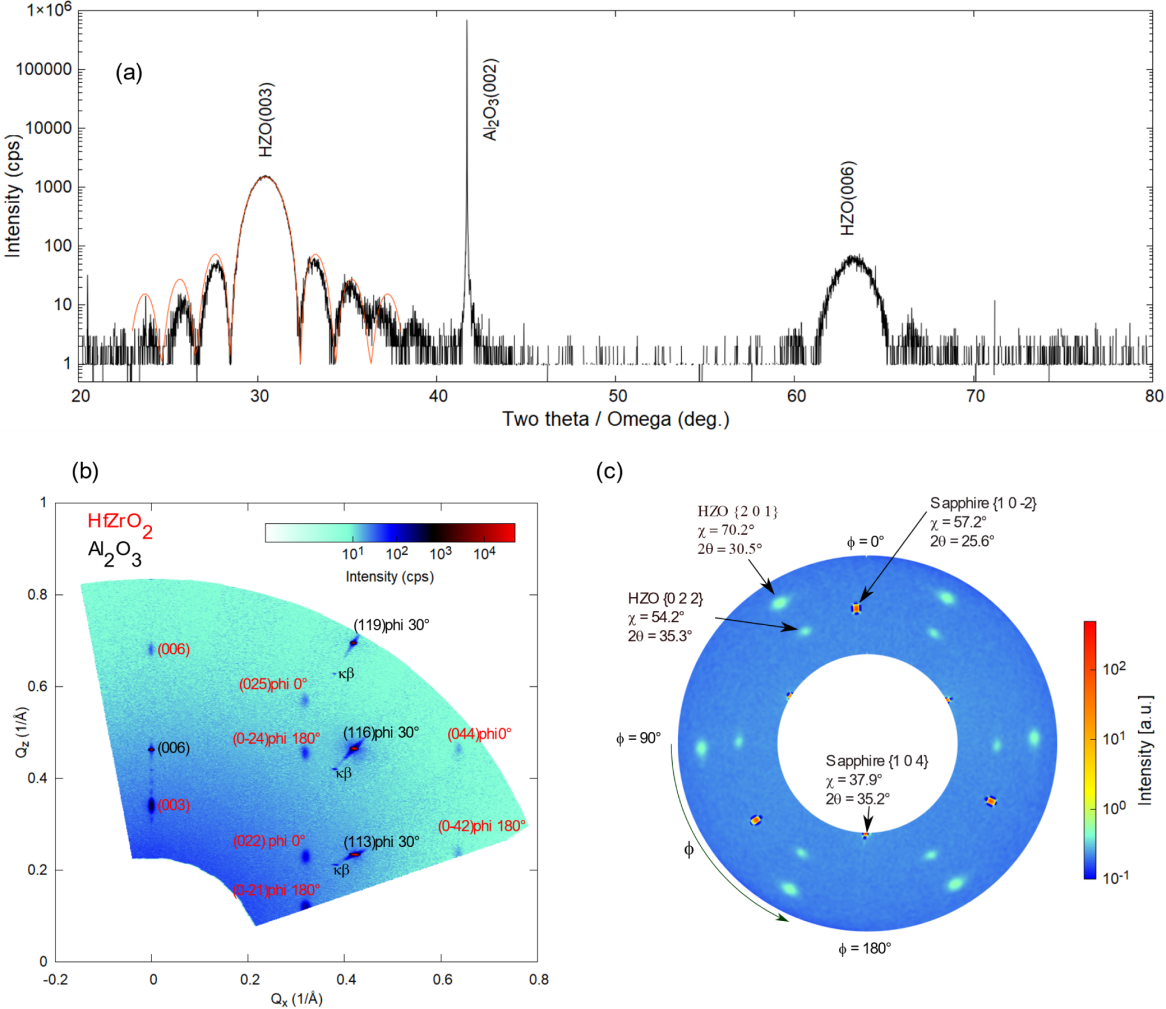
(a) High-resolution 2θ/ω scan of a 4.7 nm thick HZO film deposited on sapphire. (b) Wide range RSM of the same film on sapphire. The spots belonging to sapphire are denoted in black, whereas the spots from the HZO film are denoted in red. The angles denoted next to the HZO spots represent the in-plane rotation of the corresponding HZO domains assigned to the *R*3*m* phase. The sapphire substrate was rotated by phi = 30° during the RSM measurement. (c) Pole figure of the HZO {201}, HZO {022}, sapphire {10} and sapphire {104} spots. The radial direction represents the χ axis ranging from 0° to 90°, whereas the azimuthal direction represents the φ axis with a range from 0° to 360°.

Next, Y- and Zr-doped HfO_2_ films were deposited on trigonal Al_2_O_3_ (0001) single crystal substrates. A 5 nm thick Hf_0.5_Zr_0.5_O_2_ film deposited on Al_2_O_3_ (0001) is characterized by XRD experiments, as presented in Figure 7. The high-resolution ω-2θ scan shown in Figure 7a allowed for the precise measurement of the *c* lattice parameter, listed in Table 1, and determination of the film thickness based on the thickness-oscillation period, which in this case was 4.7 nm. A wide-range RSM shown in Figure 7b was measured at an in-plane rotation angle of φ = 30°. Thus, in addition to the specular 006 spot, the 113, 116, and 119 spots of the Al_2_O_3_ substrate are observed at this in-plane phi angle. The HZO film appears to consist of two kinds of domains, belonging to the same r-phase *R*3*m* no. 160, rotated in-plane by 180° with respect to one another and by 30° with respect to the Al_2_O_3_ crystallographic cell. The corresponding angles of these two domains, which were obtained from the simulation of the RSM are denoted next to indexed HZO spots in the figure. The pole figure of the same sample is presented in Figure 7c. The HZO {201}, HZO {022}, sapphire {10} and sapphire {104} spots from the lattice planes symmetrically equivalent are marked on the pole figure. The 2θ ranges from 25° to 37.8°.

**Table 1:** *a* and *c* Lattice Parameters of the Strained Doped HfO_2_ Films Deposited on Different systems in this Work Were Extracted from High-Resolution 2θ/ω Scans and In-Plane Scans^a^

heterostructure			*a* (Å)	*c* (Å)
8 nm HZO/LSMO/STO			7.194	8.899
7.7 nm HYO/LSMO/STO			7.170	8.953
7.6 nm HZO/LSMO/STO/Si			7.236	8.822
5.6 nm HYO/LSMO/STO/Si			7.212	8.883
6.2 nm HYO/Al_2_O_3_			7.207	8.879
2.6 nm HYO/Al_2_O_3_			7.239	8.773
4.7 nm HZO/Al_2_O_3_			7.219	8.805
HfO_2_, *R*3*m* (no. 160); *a* = *b*; α = 90°; β = 90°; γ = 120°				
atom	*x*	*y*	*z*	occupancy
Hf	0.83335	0.16665	0.25089	1
Hf	0.00000	0.00000	0.58415	1
O	0.14966	0.85034	0.15904	1
O	0.48699	0.51301	0.32788	1
O	0.00000	0.00000	0.85998	1
O	0.00000	0.00000	0.35364	1

a The crystallographic structure is the one reported by Wei et al. in ref (13) adapted to the lattice parameters measured in this work.

**Figure 8:**
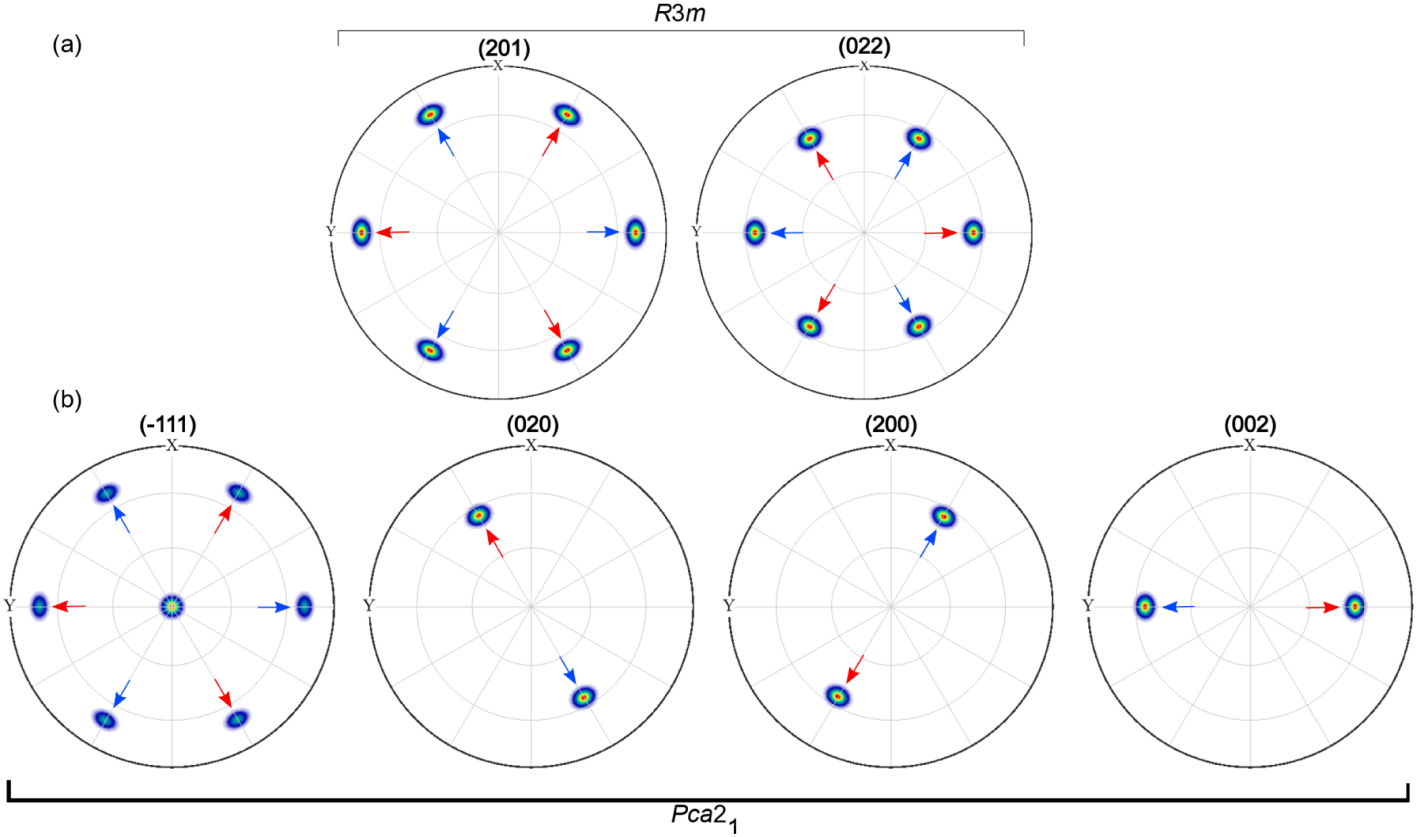
Pole figure simulation of the HZO films using MTEX. (a) Phase *R*3*m*, considering (001) out-of-plane orientation and presence of two domains (indicated by the red and the blue arrows), with an in-plane angular rotation of 180° with respect to one another. (b) Phase *Pca*2_1_ considering (111) out-of-plane orientation, and the presence of two domains, with an in-plane angular rotation of 180° with respect to one another.

The sapphire {10} and {104} spots reflect the trigonal symmetry of the phase, resulting in three spots on the pole figure for each family. The HZO {201} and HZO {022} spots should also show three spots each, according to the crystallographic symmetry of the *R*3*m* phase, but six spots are observed experimentally. This can be explained by the presence of two kinds of domains, belonging to the same trigonal phase *R*3*m*, rotated in-plane by 180° with respect to one another, as deduced for the wide-range RSM simulation discussed above. This domain configuration has been reported in prior studies on HZO films deposited on GaN and sapphire,^14^ also being imposed by the symmetry of the substrate. To verify that, a pole figure of the HZO films was simulated using MTEX, according to the proposed assumption, and the results are shown in Figure 8a. The simulation corresponds to the findings obtained from the experiments.

The simulation for the *Pca*2_1_ phase of a (111) oriented monodomain film gives three poles from the {11}, and one poles from each {020}, {002} and {200} reflection families. If one considers two (111) domains rotated in-plane by 180° with respect to one another, the simulations reproduce the observed six {111} poles, as well as the six poles of the {020}, {002}, and {200} ones, as shown in Figure 8b. Also, in this case, it is difficult to distinguish between the *R*3*m* and *Pca*2_1_ crystallographic phases from the pole figure XRD measurements.

Considering the relatively uniform intensity distribution of the reflections corresponding to a specific family of spots, one can state that the volume fraction of the two types of HZO domains appears to be equally distributed.

The measured wide-range RSM of the HZO deposited on sapphire could also be assigned to a ferroelectric orthorhombic phase like, for example, the one reported by Xu et al.^39^ With this assumption, the films are epitaxial and have an (111) out-of-plane orientation. Such an example is illustrated in Figure S2, where the observed spots in the RSM from the HfO_2_ films deposited on sapphire are assigned to the above-mentioned crystallographic phases.

**Figure 9:**
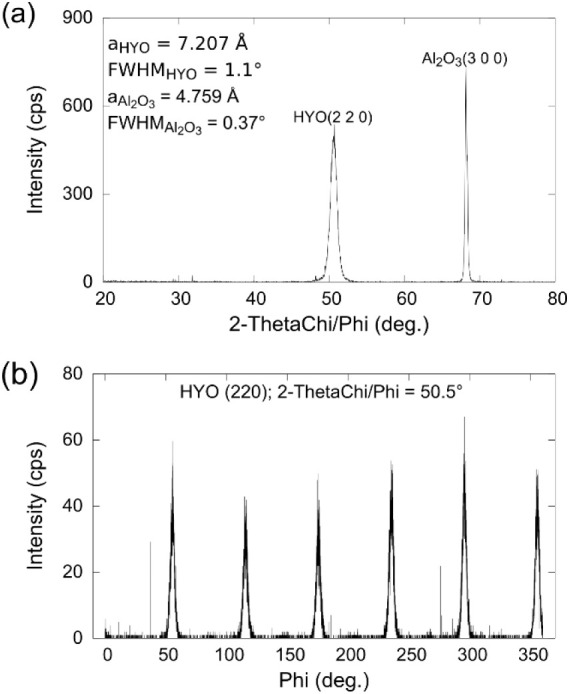
In-plane XRD of the HYO(6.2 nm)/Al_2_O_3_: (a) in-plane scan showing the HYO(220) and Al_2_O_3_ (300) reflections; the lattice parameters and the FWHM are given in the inset. (b) In-plane phi-scan of the HYO(220) region showing a 6-fold in-plane symmetry; the FWHM is 4.1°.

To precisely determine the in-plane lattice parameters of the deposited films, we performed in-plane X-ray diffraction measurements. Thus, in the case of doped hafnia films deposited on sapphire, the HYO (220) and Al_2_O_3_ (300) reflections were observed, as can be seen in Figure 9a. This means that the (220) crystallographic planes of the epitaxially doped hafnia films are parallel with the (300) planes of the underlying sapphire substrates and perpendicular to the HYO (003) planes which are parallel with the sample surface. The epitaxial relationship in this case considering the *R*3*m* crystallographic phase is [220] HZO(001) //[300] Al_2_O_3_(001). If one would consider the ferroelectric orthorhombic phase *Pca*2_1_ instead of the trigonal phase (*R*3*m*), the (111) crystallographic plane is parallel with the sample surface, whereas the (20) plane forms an angle of 89.84° with the (111) plane, very close to 90°. For this reason, based on the in-plane XRD measurements, one cannot distinguish between the ferroelectric orthorhombic phase and the rhombohedral one.

From these high-resolution in-plane scans, one can precisely determine the in-plane lattice parameter of the rhombohedral HYO films deposited on sapphire, which is 7.207 Å. Next, the 2-ThetaChi/Phi axis was fixed at the HYO(220) peak position, and a phi-scan was performed, with a complete 360° phi-axis scan range. The 3-fold in-plane symmetry expected for the rhombohedral films combined with the presence of the two 180° in-plane rotated domains is consistent with the observed six reflections spaced by 60° from this scan and with the results from the pole figures of the HZO films deposited on sapphire, discussed above. The in-plane phi scan shown in Figure 9b is equivalent to an in-plane rocking curve, and the width of the reflections gives us a measure of the in-plane mosaicity. In the case of 6.2 nm HYO films deposited on sapphire, the FWHM of the in-plane rocking curve is 4.1°.

A table with the precisely measured lattice parameters of the epitaxially doped hafnia films, extracted from high-resolution specular and in-plane scans, for films deposited on different heterostructures/substrates with different film thicknesses is shown below. The *R*3*m* crystallographic phase including the structure parameters is taken from Wei et al.^13^

##### 3.1.3.2 Transmission Electron Microscopy Characterization

**Figure 10:**
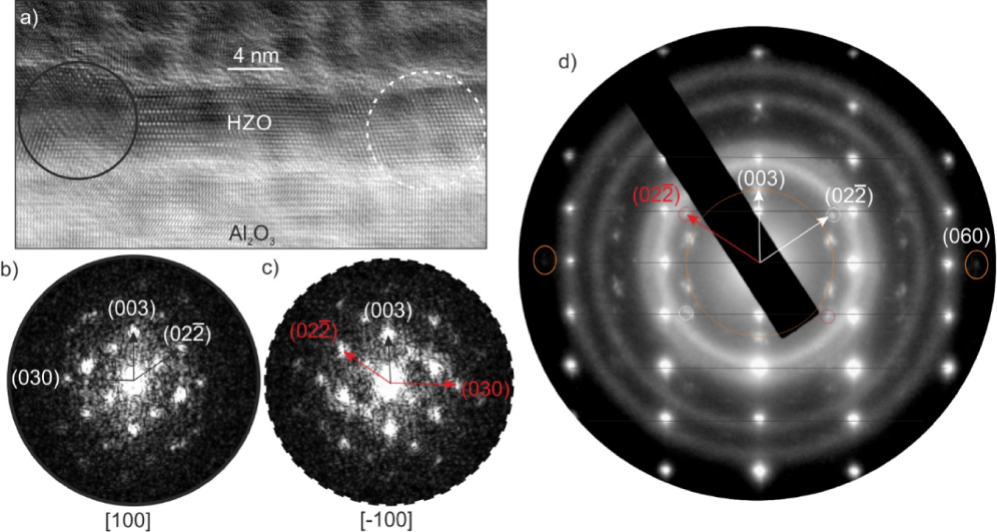
(a) HRTEM micrograph of Al_2_O_3_ in [210] ZA and HZO in [100] ZA. Two different domains were found via FFT analysis in (b) and (c); again, the [100] ZA is mirrored at the (003) plane. The SAED pattern in (d) shows both domains, and the Al_2_O_3_ [210] ZA is indicated by vertical black lines. In this system, the (003) reflections are rather weak compared to the STO/LSMO system, and the (060) reflection is visible in-plane. In FFTs even the (030) planes. The diffraction rings originate from the Pt coating which was used to protect during FIB preparation.

The TEM analysis of HZO on sapphire is shown in Figure 10. HZO on sapphire is divided into two domains, both growing in the [003] direction (considering the *R*3*m* phase) with a 2-fold twin rotation as shown in the HRTEM micrograph and the corresponding FFTs in Figure 10b,c. On sapphire, however, no additional growing direction was observed. In comparison to HZO on an STO substrate, the out-of-plane (003) reflections in the SAED pattern have a weak intensity, while for the in-plane (030) reflection, a higher intensity is observed. For both substrates, the intensities of the diffraction pattern is comparable. This may suggest a stronger in-plane epitaxial relation of (20) of Al_2_O_3_ and (030) or (300) of HZO in comparison to those deposited on STO substrates.

### 3.2 Electrical Characterization

**Figure 11:**
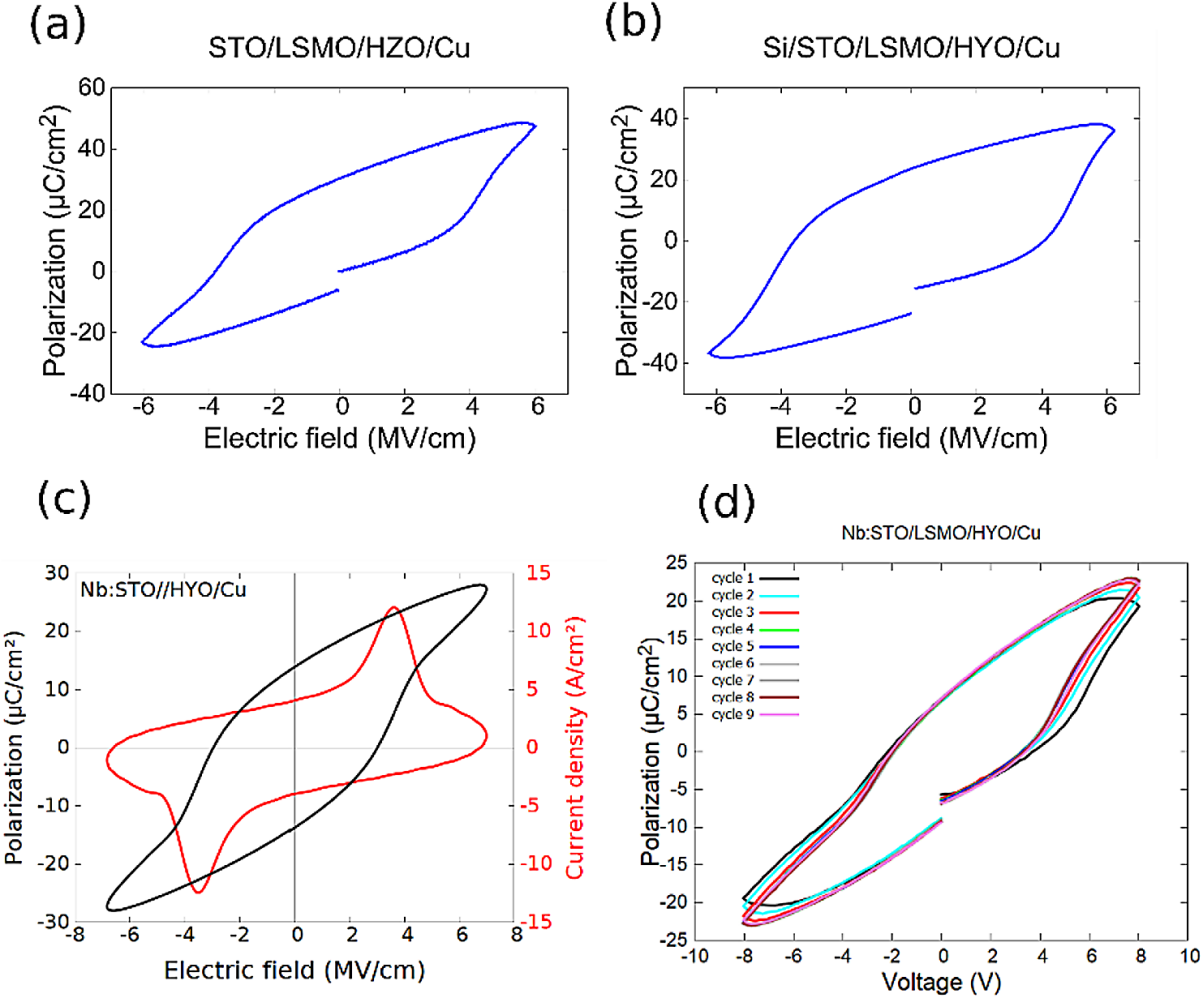
Ferroelectric characterization. Dynamic ferroelectric polarization hysteresis loops of the (a) Cu/Hf_0.5_Zr_0.5_O_2_/La_0.7_Sr_0.3_MnO_3_/SrTiO_3_, (b) Cu/Hf_0.93_Y_0.07_O_2_/La_0.7_Sr_0.3_MnO_3_/SrTiO_3_/Si, and (c) Cu/Hf_0.93_Y_0.07_O_2_/La_0.7_Sr_0.3_MnO_3_/Nb:SrTiO_3_ capacitors. (d) “Wake-up” effect investigation on a pristine ferroelectric capacitor.

Ferroelectric dynamic polarization hysteresis loops of the HZO films deposited on STO (substrate)/LSMO and Si (substrate)/STO/LSMO were measured to test the ferroelectric behavior of the doped hafnia films. The capacitor devices have a Cu top electrode, and the device area is 225 μm^2^ for the films deposited on STO substrates and 50 μm^2^ for those deposited on the Si substrate. No correction or leakage current compensation was used for the dynamic polarization hysteresis loop measurements. The results are listed in Figure 11. All of the measured samples show ferroelectricity, with a remanent polarization ranging from 14 to 26 μC/cm^2^. These polarization values are comparable with those reported in the literature for epitaxial doped HfO_2_ films.^13,18^

The “wake-up” effect in these ferroelectric capacitors was investigated by acquiring the first 9 consecutive *P*–*V* cycles on a pristine capacitor, as presented in Figure 11d. The samples show ferroelectricity from the first *P*–*V* cycle, and after the third cycle, there is no significant change in the shape of the *P*–*V* ferroelectric loops.

## 4 Conclusions

In conclusion, ultrathin epitaxial ferroelectric Y- and Zr-doped HfO_2_ films are successfully deposited on three different systems: La_0.7_Sr_0.3_MnO_3_-buffered SrTiO_3_ substrates, La_0.7_Sr_0.3_MnO_3_ SrTiO_3_-buffered Si (100) wafers, and trigonal Al_2_O_3_ substrates. To distinguish between the commonly reported orthorhombic phase and other possible ferroelectric polymorphs like the rhombohedral *R*3*m* phase employing XRD methods, combining θ*-*2θ symmetric scans, in-plane scans, wide-range reciprocal space maps, and pole figure measurements turned out to be a difficult task due to the structural similarities of the polymorphs. However, extensive XRD characterization indicates that the majority of the investigated films consist of the trigonal phase (*R*3*m*, no. 160) with *c*-axis orientation. The films deposited on La_0.7_Sr_0.3_MnO_3_-buffered SrTiO_3_ substrates and La_0.7_Sr_0.3_MnO_3_ SrTiO_3_-buffered Si (100) wafers consist of four kinds of domains, rotated in-plane with 90° with respect to one another, whereas the films deposited on sapphire consist of two kinds of domains, rotated in-plane with 180° with respect to one another, the domains having an equal volume-fraction. TEM analysis supports these conclusions. However, for the doped HfO_2_ films deposited on La_0.7_Sr_0.3_MnO_3_-buffered SrTiO_3_ substrates, a minority growing direction ([22]) could be detected by TEM, whereas only the majority [003] growing direction was observed for those deposited on sapphire. By the analysis of HRTEM micrographs of a plan-view of doped hafnia films deposited on STO-buffered Si, a domain size of about 10 nm could be estimated. The ferroelectric characterization of capacitors based on Y- or Zr-doped HfO_2_ films deposited on La_0.7_Sr_0.3_MnO_3_-buffered SrTiO_3_ substrates and SrTiO_3_-buffered Si substrates show a remanent polarization ranging from 15 μC/cm^2^ to 26 μC/cm^2^ with no “wake-up” effects.

## Supporting Information Available

A wide-range RSM of the HZO/LSMO/STO heterostructure at a φ angle of 45°; wide-range RSM assigned to the *R*3*m* and *Pca*2_1_ phases; the explanation for the shifts in the φ angle observed in the measured wide range pole figures; HRTEM micrograph of the HYO/LSMO/STO/Si thin film stack; simulated pole figures for the *R*3*m* and *Pca*2_1_ phases at a 2-theta of about 50°, and a chi angle of 35° (PDF)

## Abbreviations

PLD: pulsed laser deposition
HZO: Hf_0.5_Zr_0.5_O_2_
HYO: Hf_0.93_Y_0.07_O_2_
LSMO: La_0.7_Sr_0.3_MnO_3_
STO: SrTiO_3_
