# Supplementary material for: Distinguishing the Rhombohedral Phase from Orthorhombic Phases in Epitaxial Doped HfO2 Ferroelectric Films
Source: ACS Appl Mater Interfaces. 2024 Aug 5;16(32):42534–45. doi: 10.1021/acsami.4c10423 (PMC11331437; doi:10.1021/acsami.4c10423)
Supplement: Supplementary file 1 — am4c10423_si_001.pdf [file am4c10423_si_001.pdf]

Supporting information

Distinguishing the Rhombohedral Phase from Orthorhombic Phases in Epitaxial Doped HfO_2 Ferroelectric Films

Adrian Petraru[1], Ole Gronenberg[2], Ulrich Schürmann[2,4], Lorenz Kienle[2,4], Ravi Droopad[3], Hermann Kohlstedt[1,4]*

[1]Nanoelectronics, Institute of Electrical Engineering and Information Engineering, Kiel University, Kiel D-24143, Germany.

[2]Institute for Materials Science – Synthesis and Real Structure, Faculty of Engineering, Kiel University, Kiel D-24143, Germany

[3]Ingram School of Engineering, Texas State University, San Marcos, Texas 78666, USA

[4] Kiel NanoSurface and Interface Science KiNSIS, Kiel University, Christian-Albrechts-Platz 4, Kiel D-24118, Germany.

* E-mail: apt@tf.uni-kiel.de

Additional wide-range RSM of the HZO/LSMO/STO heterostructure at a φ angle of 45°.

Figure S1 shows an additional wide-range RSM of the HZO/LSMO/STO heterostructure. Here, at this particular φ angle of 45°, the 111, 112, 113, and 221 reflections of the STO/LSMO are also present in the RSM.

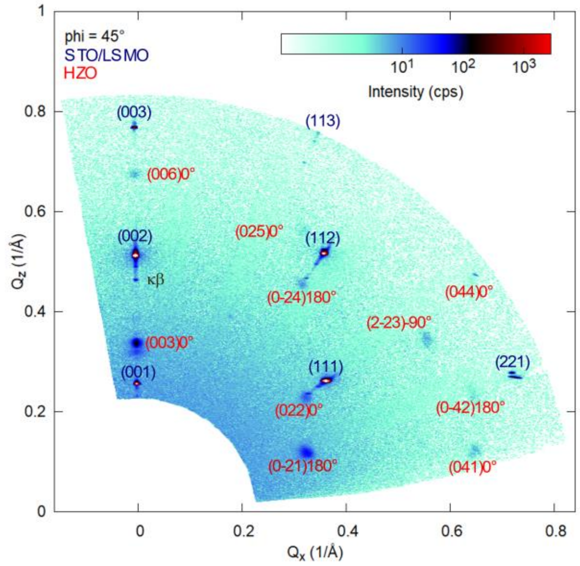

Figure S1. Wide-range RSM of an 8 nm thick HZO film on LSMO/STO(substrate). The measurement was performed at an angle φ = 45° with respect to the in-plane *a*-direction of the STO substrate. The observed spots from the HZO film are assigned to the ferroelectric *R*3*m* phase and marked in red, the angle represents the HZO angle of the contributing domains. The STO/LSMO spots are marked in dark blue.

Wide range RSM assigned to the *R*3*m* and *Pca*2₁ phases.

Figure S2 illustrates how the measured wide-range RSM of a 4.7 nm thick HZO film deposited on sapphire can be attributed to both *R*3*m* and *Pca*2_1 phases, so one cannot differentiate between these two phases based on this type of measurement. The same is valid for the doped hafnia films deposited on LSMO-buffered STO.

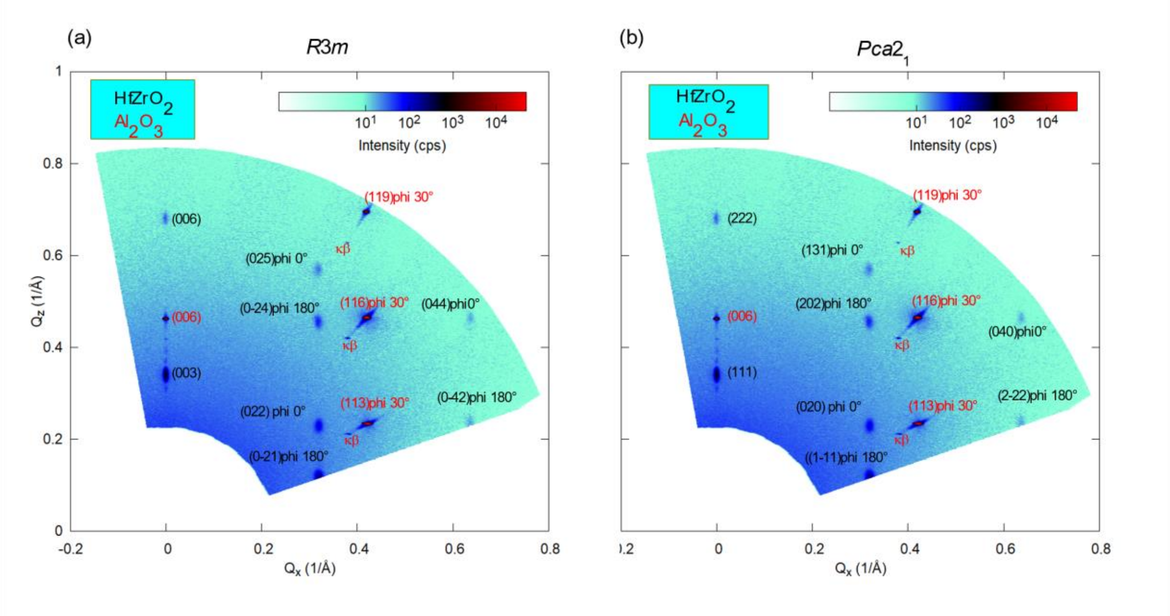

Figure S2. Wide-range RSM of a 4.7 nm thick HZO film deposited on sapphire. The observed spots from the HZO film could be assigned to the ferroelectric (a) *R*3*m* phase, but also to (b) *Pca*2_1 orthorhombic phase. Both phases consist of two types of domains with 0° and 180° in-plane orientation. The spots belonging to the sapphire are denoted in red, whereas the spots from the HZO film are denoted in black.

The explanation for the shifts in φ angle observed in the measured wide range pole figures for spots with a (large) separation in 2-theta and chi.

The spots of $\{10\bar{2}\}$ and $\{104\}$ families of the Al_2O_3 single-crystal substrates in the pole figure should have an angular shift of 60° between them, as shown in Figure S3, from the MTEX simulations. However, there is an additional angular shift that appears in the measured data from Figure 7c for the above-mentioned spots. This additional shift of about 5° appears for spots measured at different 2θ and χ angles on the same pole figure and originates from the instrumentation, as illustrated for the $\{10\bar{2}\}$ and $\{104\}$ sapphire substrates spots. Such shifts are also visible in the case of HZO $\{201\}$ and HZO $\{022\}$ in Figure 2b, Figure 5b, and Figure 7c.

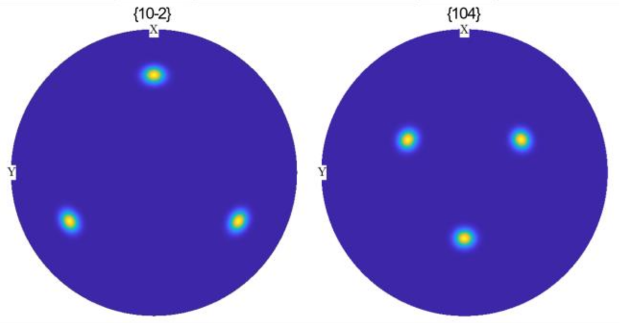

Figure S3. Pole figure simulation of the Sapphire $\{10\bar{2}\}$ and $\{10\bar{4}\}$. Each family of spots contributes with three spots spaced by 120°, and the angular shift between the two families of spots is 60°.

Additional HRTEM micrograph of the HYO/LSMO/STO/Si thin film stack.

The diffraction pattern in the FFT in Figure S4b is either formed by a rotational Moiré or by double diffraction from the intense Si/STO reflection. However, in both cases, the pattern originates from the HYO film. The filtered FFT in Figure S4c was used to determine a rough estimate of the grain size by calculating the mean of 20 grains (the number of clearly distinguishable grains). By this method, a grain size of 10 nm ±3 nm was measured.

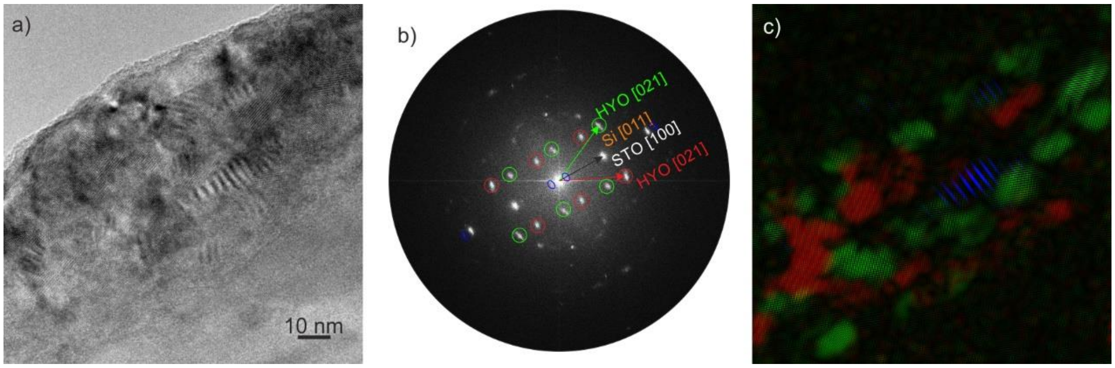

Figure S4. (a) HRTEM micrograph of the thin film stack tilted away from [100] zone axis of the substrate with Moiré pattern. The FFT in (b) shows the [011] direction of Si and [100] of STO. Filtering the red, green, and blue regions in the FFT reveals the different orientations in the HYO film. The inverse FFT of the filtered regions is shown in (c). The intensity marked in blue represents the minority of the HYO oriented in plane with STO [100].

Simulated pole figures for the *R*3*m* and *Pca*2$_1$ phases at a 2-theta about 50°, and a chi angle of 35°.

The simulated pole figures from Figure S5 help the reader to understand the associations of the scans from Figure 3c to particular reflections and domains, considering both *R*3*m* and *Pca*2$_1$ phases. The spots corresponding to the four domains are indicated by black, red, blue, and green arrows, respectively.

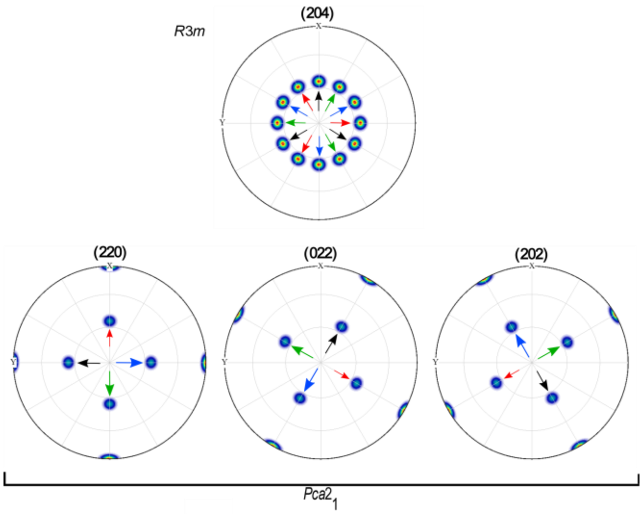

Figure S5. Pole figure simulation of the HZO films at 2-theta about 50°; the spots are at a chi angle of 35°. Top: *R*3*m* phase, considering (001) out-of-plane film orientation and presence of four domains, with an in-plane shift of 90° with respect to one another. Bottom: *Pca*2$_1$ phase considering (111) out-of-plane film orientation, the presence of four domains, with an in-plane shift of 90° with respect to one another, and considering the {220}, {022}, and {202} reflections. The black, red, blue, and green arrows indicate the four domains with the families of spots associated with them.
